# Lassa Fever, Nigeria, 2005–2008

**DOI:** 10.3201/eid1606.100080

**Published:** 2010-06

**Authors:** Deborah U. Ehichioya, Meike Hass, Stephan Ölschläger, Beate Becker-Ziaja, Christian O. Onyebuchi Chukwu, Jide Coker, Abdulsalam Nasidi, Osi-Ogbu Ogugua, Stephan Günther, Sunday A. Omilabu

**Affiliations:** University of Lagos, Lagos, Nigeria (D.U. Ehichioya, S.A. Omilabu); Bernhard Nocht Institute for Tropical Medicine, Hamburg, Germany (M. Hass, S. Ölschläger, B. Becker-Ziaja, S. Günther); Ebonyi State University Teaching Hospital, Abakaliki, Nigeria (C.O. Onyebuchi Chukwu); Federal Ministry of Health, Abuja, Nigeria (J. Coker, A. Nasidi); National Hospital, Abuja (O.-O. Ogogbu)

**Keywords:** Lassa fever, viruses, hemorrhagic fevers, arenavirus, Nigeria, letter

**To the Editor:** Lassa fever affects ≈100,000 persons per year in West Africa (*1*). The disease is caused by Lassa virus, an arenavirus, and is associated with bleeding and organ failure. The case-fatality rate in hospitalized patients is 10%–20%. The reservoir of the virus is multimammate mice (*Mastomys natalensis*). Investigations in the 1970s and 1980s pointed to the existence of 3 disease-endemic zones within Nigeria: the northeastern region around Lassa, the central region around Jos, and the southern region around Onitsha (*2*,*3*). The current epidemiologic situation is less clear because no surveillance system is in place.

In 2003 and 2004, we conducted a hospital-based survey in Irrua, which demonstrated ongoing transmission of the virus in Edo State, Nigeria (*4*). Since then, laboratory capacity at the University of Lagos for diagnosing Lassa fever has been improved and used for small-scale passive surveillance in other parts of the country. Public health officials or hospital staff reported suspected cases. Blood samples were sent to Lagos, or staff from Lagos collected samples on site. Confirmatory testing, sequencing, and virus isolation were performed at the Bernhard Nocht Institute for Tropical Medicine in Hamburg, Germany. Primary testing was done by reverse transcription–PCR (RT-PCR) that targeted the glycoprotein (GP) gene (*5*,*6*). An RT-PCR that targeted the large (L) gene was used as a secondary test (*7*), and PCR products were sequenced. Serologic testing for Lassa virus–specific immunoglobulin (Ig) G and IgM was performed by immunofluorescent antibody test using Vero cells infected with Lassa virus. Virus isolation with Vero cells was conducted in the BioSafety Level 4 laboratory in Hamburg.

From 2005 through 2008, 10 cases of Lassa fever were confirmed by virus detection (cases 3–10) or implicated by epidemiologic investigation and serologic testing (cases 1 and 2) (Appendix Table). Case-patients 1–4 were involved in a nosocomial outbreak that occurred in February 2005 at the Ebonyi State University Teaching Hospital (EBSUTH) in Abakaliki. Retrospective investigation suggests the following transmission chain. The presumed index case-patient was a male nurse living in Onitsha, who became ill on January 21, 2005, and traveled ≈200 km to EBSUTH for better medical treatment. The detection of Lassa virus–specific IgM during his convalescent phase indicates that he had Lassa fever. The second case-patient was a female nurse who had contact with the index case-patient on February 4. She was admitted on February 7 and died 6 days later. Her clinical features were compatible with Lassa fever, but laboratory confirmation is lacking because specimens were not collected. Two additional case-patients among hospital staff (case-patients 3 and 4) were seen on February 21; each had had contact with case-patient 2. Case-patient 3 took care of case-patient 2 and slept in the same room with her for 4 days. Lassa fever was confirmed in case-patients 3 and 4 by RT-PCR as well as by IgM and IgG seroconversion in the surviving patient (case-patient 3). Case-patient 4, a pregnant nurse, had a spontaneous abortion and died on day 9 of hospitalization. Sequencing the GP and L gene PCR fragments showed that case-patients 3 and 4 were infected with the same virus strain (100% identity). In March and April 2005, blood was collected from 50 hospital staff members (including those who had had contact with the case-patients) and screened for Lassa virus–specific IgM and IgG. No positive blood samples were found, which indicated that no additional staff members were involved in the outbreak.

Case-patients 5 and 6 were admitted to EBSUTH in 2008 on January 17 and March 5, respectively. Both were medical doctors, one at a local hospital and the other at EBSUTH, and both died. Encephalopathy with generalized seizures and loss of consciousness preceded death in both cases. The source of infection is unknown, although it is likely that they became infected while they treated patients without knowing they had Lassa fever. In agreement with the epidemiology, the viruses from the 2 patients were similar, though not identical (89% and 87% identity in the GP and L genes, respectively).

Cases 7 to 10 occurred in Abuja and Jos from December 2007 through March 2008. Healthcare workers appeared not to be involved, and no molecular epidemiologic evidence indicated that transmission occurred among the 3 case-patients from Jos (94–97% and 90–94% identity in the GP and L genes, respectively).

In conjunction with our previous report (*4*), the cases presented here demonstrate current Lassa fever activity in the states of Edo, Ebonyi, Federal Capital Territory, and Plateau. These findings correspond to early reports on Lassa fever in southern and central parts of Nigeria. That healthcare workers are still at as high a risk of contracting and dying from the disease as they were 20 years ago (*8*) is alarming.

A key to solving this problem would be the establishment of diagnostic facilities that can provide rapid molecular testing at referral centers in the disease-endemic zones. This testing would facilitate appropriate case and contact management, including early treatment and postexposure prophylaxis with ribavirin, and eventually raise awareness that Lassa fever should be considered in every severe febrile illness in these regions.

## Supplementary Material

Appendix Table Characteristics of Lassa fever cases, Nigeria, 2005- 2008*
