# Supplementary material for: Lassa Fever, Nigeria, 2005–2008
Source: Emerg Infect Dis. 2010 Jun;16(6):1040–1. doi: 10.3201/eid1606.100080 (PMC3086228; doi:10.3201/eid1606.100080)
Supplement: Appendix Table — Characteristics of Lassa fever cases, Nigeria, 2005- 2008* [file 10-0080-appT-s1.pdf]

Appendix Table. Characteristics of Lassa fever cases, Nigeria, 2005– 2008*

Locality (hospital)	Case no.	Date of admission	Age, y/sex	Symptoms	Treatment	Outcome	HCW	GP and L gene RT-PCR	IgM IFAT†	IgG IFAT†	Virus isolation	Lassa virus strain (GenBank accession nos.)‡
Abakaliki, Ebonyi State (EBSUTH)	1	2005 Feb 4	40/M	Fever	Antimicrobial drugs, antimalaria prophylaxis	Survived	Nurse	Neg§	1:160§	>1:80§	ND	–
	2	2005 Feb 7	54/F	Fever, vomiting, diarrhea, respiratory distress, oliguria	Antimicrobial drugs, corticoid	Died Feb 13	Nurse/contact to case 1	ND	ND	ND	ND	–
	3	2005 Feb 21	35/F	Fever, severe weakness	NA	Survived	Nurse/contact to case 2	Pos Neg§	Neg 1:640§	Neg >1:5120§	Neg	Nig05-SE40 (GU481058, GU481059)
	4	2005 Feb 21	36/F	Fever, vomiting, nausea, spontaneous abortion, shock	Antimicrobial drugs, tracheotomy	Died Mar 1	Nurse/contact to case 2	Pos	Neg	Neg	Neg	Nig05-043 (GU481056, GU481057)
	5	2008 Jan 17	38/M	Fever, vomiting, diarrhea, abdominal tenderness, anuria, generalized seizure, unconsciousness	Antimicrobial drugs, antimalaria prophylaxis, dobutamine, dopamine, furosemide	Died Jan 23	Doctor	Pos	Neg	Neg	Neg	Nig08-03 (GU481066, GU481067)
	6	2008 Mar 5	38/M	Fever, vomiting, hiccups, bloody diarrhea, abdominal tenderness, generalized seizure, unconsciousness	Antimicrobial drugs, antimalaria prophylaxis, assisted ventilation	Died Mar 11	Doctor	Pos	1:20	1:20	Pos	Nig08-04 (GU481068, GU481069)
Abuja, FCT State (NHA)	7	2008 Jan 2	37/M	Fever, vomiting, diarrhea, abdominal tenderness, confusion, unconsciousness	Antimicrobial drugs, ribavirin on day of death	Died Jan 7	No	Pos	1:20	1:20	Neg	Nig08-02 (GU481063 to GU481065)
Jos, Plateau State	8	2007 Dec	19/F	NA	NA	Died	NA	Pos	Neg	Neg	Neg	Nig07-05 (GU481060 to GU481062)
	9	2008 Feb	30/M	NA	NA	Died	No	Pos	1:1250	1:80	Pos	Nig08-A18 (GU481070, GU481071)
	10	2008 Feb	28/F	NA	NA	Survived	No	Pos	1:80	1:80	Pos	Nig08-A19 (GU481072, GU481073)

*HCW, health care worker; GP, glycoprotein; L, large; Ig, immunoglobulin; RT-PCR, reverse transcription PCR; IFAT immunofluorescent antibody test; EBSUTH, Ebonyi State University Teaching Hospital; pos, positive; neg, negative; ND, not done; NA, data not available; FCT, Federal Capital Territory; NHA, National Hospital Abuja.
†Titer of IFAT (cut-off 1:20).
‡Partial GP and L gene sequences were obtained by sequencing the fragments amplified by the diagnostic RT-PCRs. Additional nucleoprotein gene sequences were generated for Nig07-05 and Nig08-02, and strains isolated in cell culture were completely sequenced (D. Ehichioya, unpub. data).
§Convalescent-phase serum sample.
